# First glanders cases detected in Nepal underscore the need for surveillance and border controls

**DOI:** 10.1186/s12917-022-03233-4

**Published:** 2022-04-06

**Authors:** Koirala P, Maharjan M, Manandhar S, Pandey KR, Deshayes T, Wang G, Valvano MA, Laroucau K

**Affiliations:** 1 Central Veterinary Laboratory, Kathmandu, Nepal; 2 Veterinary Laboratory, Surkhet, Nepal; 3 ANSES, Laboratory for Animal Health, Bacterial Zoonosis Unit, European and OIE Reference Laboratory for Glanders, Paris-Est University, Maisons-Alfort, France; 4 Wellcome-Wolfson Institute for Experimental Medicine, Queen’s University Belfast, Belfast, UK

**Keywords:** *Burkholderia mallei*, Equids, India

## Abstract

**Background:**

Glanders is a transmissible zoonotic disease caused by *Burkholderia mallei* that infects equids and humans. No glanders cases in equids were reported so far in Nepal.

**Case presentation:**

Following suspected glanders in animals with clinical signs in different regions in Nepal, serum samples were tested by CFT, ELISA and Luminex® tests. Two horses and a mule tested positive for glanders by all tests, while two other equids only tested positive by ELISA and Luminex®. Analysis of swabs and pus samples by a PCR system targeting *B. mallei* confirmed the presence of the bacterium in the samples collected from the 3 equids that yielded positive results in all serological tests. Genotyping of the three PCR positive samples with a SNP-based method identified a genotype closely related to the *B. mallei* strains circulating in India.

**Conclusion:**

Confirmation of glanders cases underscores the need of implementing a surveillance program in Nepal and a strict control of the animal movement across the borders.

## Background

Glanders is an infectious disease caused by *Burkholderia mallei.* This zoonotic bacterium primarily infects equids [1]. Several outbreaks of glanders in equids have been recently reported in South Asia, the Middle East, and South America (Brazil) [2]. Clinical and laboratory diagnosis of glanders is difficult since limited clinical signs are expressed in the early stage of infection. Symptoms of *B. mallei* infection include nasal discharge, pneumonia, and ulcerating nodular lesions on the skin. Discharges from the respiratory tract and skin are infectious. Transmission between animals is facilitated by close contact, inhalation, ingestion of contaminated materials (e.g., from infected feed and water troughs), or by inoculation (e.g., via a harness). Diagnostic methods of glanders include immunological tests such as complement fixation test (CFT) or ELISA and/or allergic reaction (malleinization), as well as direct tests such as bacteriological isolation and molecular tests. For *B. mallei* typing, the high-resolution melting PCR (HRM-PCR) technique targeting single nucleotide polymorphisms (SNPs) allows categorization of strains into three lineages (L1 to L3), as well as branches, sub-branches, and clusters with geographic specificities [3–5].

### Case presentation

**Fig. 1:**
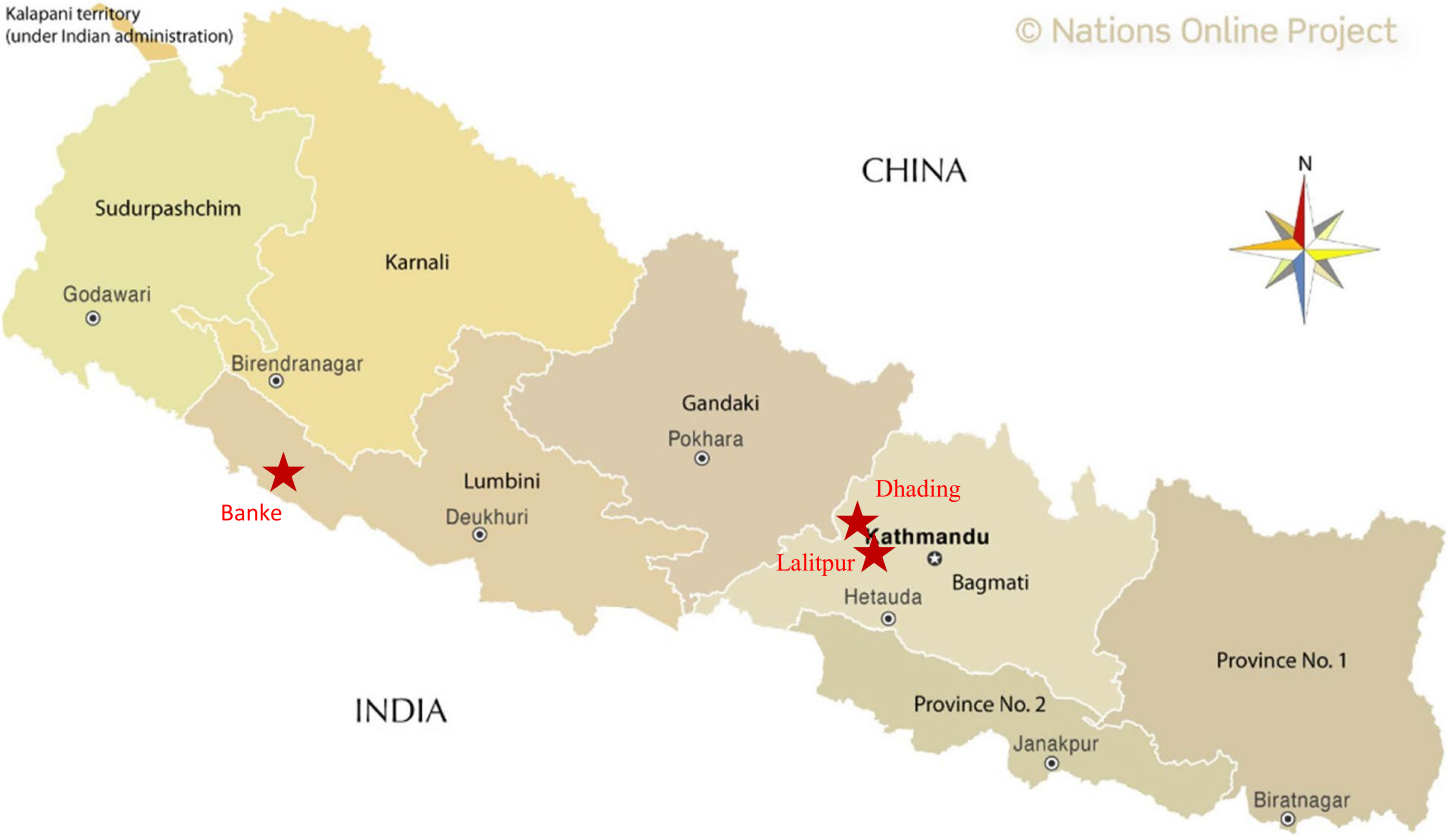
Map showing the geographical locations of the glanders-affected districts in Nepal, between November and December 2020 (map source: © Nations Online Project, https://www.nationsonline.org/oneworld/map/nepal-administrative-map.htm)

**Fig. 2:**
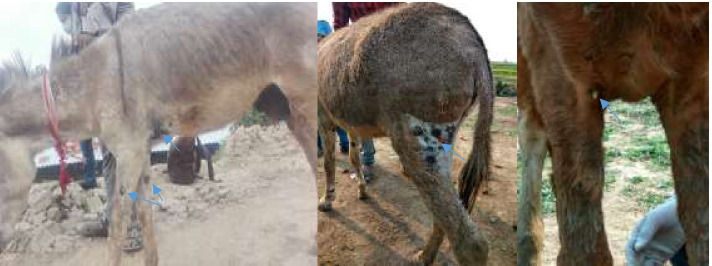
Lesions observed in mules (pus filled nodules and thick yellowish pus discharge from nodules)

In November 2020, equids in Banke district of mid-western Nepal developed clinical signs and symptoms like high grade fever (up to 40-41^0^C), labored breathing, dry cough, loss of appetite, lameness, thick mucopurulent yellowish nasal discharge, pus filled nodules on different parts of body, especially on thigh area. Later, in December 2020, similar clinical symptoms and signs were observed in mules in Dhading and Lalitpur districts of the Bagmati Province. The death of several equids was also reported in Nepalgunj, Lumbini province (Fig. 1). Available clinical surveillance data are reported in Table 1. Sticky yellowish pus discharge from ulcerated nodules and scabs was noticed on some of the infected equids (Fig. 2). Mules showed more severe symptoms than horses. All the infected animals were isolated and given symptomatic treatment. In most cases, symptoms relapsed after some time, became more severe and animals died of the disease. There is no policy to euthanize glanders infected animals in Nepal.

**Table 1:** Total risks population reported morbidities and mortalities

**Districts**	**Administrative Region**	**Species**	**Total population**^**a**^	**Susceptible cases**	**Cases**	**Deaths**
**Lalitpur**	Bagmati Province	Equine	250	10	2	0
**Dhading**	Bagmati Province	Equine	293	3	1	0
**Banke**	Lumbini Province	Equine	2021	77	24	16

^a^Source: Statistical Information of Nepalese Agriculture 2019-20, Page no. 91, https://www.moald.gov.np/publication/Agriculture%20Statistics

Separate data for horses, mules and donkeys aren’t available. Susceptible cases were asymptomatic equines belonging to the same farms in which cases were found. Cases were equids that developed signs and symptoms of glanders

For diagnosis and further investigations, sera and tissue samples were collected from three horses and two mules, each from different owners. One horse (L/157) came from the Lalitpur district, while the four other equids came from the Banke district (B/113, B/115, B/117 and B/120).

Sera from these five equids were analyzed with five different serological tests: (i) CFT [6], (ii) ID Screen® Glanders indirect ELISA (IdVet, France) [7], (iii) GLANDA ELISA (IdVet, France) based on two recombinant proteins [8], (iv) Luminex® bead-based assay targeting Hcp1 and GroEL proteins [9], and (v) ELISA based on a glycoengineered protein of *Burkholderia* recently validated for glanders diagnosis (Glyco ELISA) [10]. Two horses (L/157, B/120) and one mule (B/113) were positive by all tests and positive or undetermined results were obtained for all animals with ID Screen® Glanders indirect ELISA*,* GLANDA ELISA and Luminex® tests. The CFT and the ELISA test based on a glycoengineered protein identified fewer positive samples (Table 2).

**Table 2:** Serological results from the five equids tested with the complement fixation test, ELISA and Luminex^®^ methods

				**ID Screen ® Glanders indirect ELISA**^**a**^		**GLANDA ELISA**^**b**^		**Luminex – Hcp1**		**Luminex - GroEL**		**Glyco Elisa**^**c**^	
		CFT		Cut-off > 50 (40<S/*P* >= 50 : undet.)		cut-off > 50		cut-off > 43		cut-off > 45		cut-off > 40	
**Animal ID**	**species**	Titer	Result	%S/P	Result	%S/P	Result	%S/P	Result	%S/P	Result	%S/P	Result
L/157	Horse	**4441**	P	119	P	281	P	121	P	123	P	150	P
B/113	Mule	**444443**	P	117	P	301	P	100	P	110	P	164	P
B/115	Mule	0	n	80	P	285	P	62	P	90	P	9	n
B/117	Horse	0	n	47	U	288	P	146	P	45	U	2	n
B/120	Horse	**444444**	P	129	P	288	P	145	P	147	P	193	P

^a^ELISA test based on a semi-purified fraction of *B.* mallei, ^b^ELISA test based on a recombinant protein of *B.* mallei, ^c^ELISA test based on a glycoengineered antigen of Burkholderia

*P* Positive, *U* Undetermined, *n* Negative

For CFT, result were expressed as the intensity of hemolysis inhibition (0=0%, 1=25%, 2=50%, 3=75%, and 4=100%) for reach serum dilution (1/5, 1/10, 1/20, 1/40 and 1/80)

For ELISA and Luminex methods, results were expressed as S/P =((Sample – Negative control)/(Positive control - Negative control))^*^ 100. Cut-off values for each test are mentioned

In parallel, nasal or pus swabs were collected from these five animals. Samples were submitted to DNA extraction and PCR amplifications as previously described [4]. Briefly, after DNA extraction with the High Pure PCR Template Preparation Kit (Roche, Meylan, France), DNA was amplified by real-time PCR using four different PCR systems: *fliP* (specific for *B. mallei*), *orf11* (specific for *B. pseudomallei*), and *aroA* (specific for the *B. pseudomallei* complex). All samples with a quantification cycle (Cq) over 39 were considered as negatives. Both *aro*A and *fli*P PCR detected a positive signel for the three equids (L/157, B/113, B/120) that were positive by all serological tests (Table 3).

**Table 3:** Summary of the molecular analyses conducted on samples collected from L/157, B/113, and B/120

			real-time PCR			PCR-HRM clustering
			*B. pseudomallei complex*	*B.mallei fliP*	*B. pseudomallei*	
**Horse Id**	**Sample**	IPC	*aroA*	*fliP*	***orf11***	
		(Cq value)	(Cq value)	(Cq value)	(Cq value)	
L/157	swab	30.3	31.6	29.4	-	L2B2s B2 India – Group_2 (large)
B/113	swab	30	37.6	34.2	-	L2B2s B2 India – Group_2 (large)
B/115	swab	30	*40*^*a*^	-	-	/
B/117	swab	30.2	-	-	-	/
B/1	pus	32	35.5	33.3	-	L2B2s B2 India – Group_2 (large)

*IPC* Internal control with exogen DNA, - (negative), / (not done), *PCR-HRM* High-resolution melting PCR analysis for the genotyping of *Burkholderia mallei*

^a^value beyond the cut-off (sample considered as negative)

**Fig. 3:**
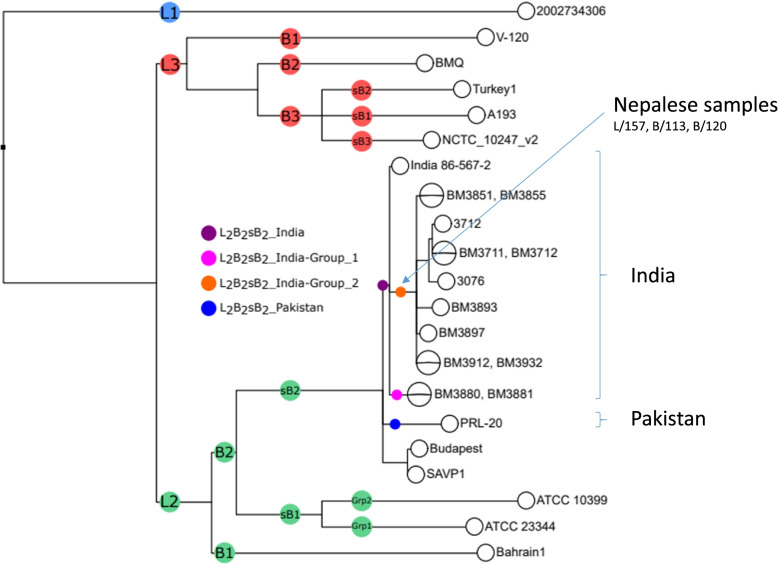
A SNP tree of *Burkholderia mallei* incorporating strains from India and Pakistan within the L2B2sB2 branch (5). PCR HRM clustering results for samples L/157, B/113, B/120 in L2B2sB2_India _Group 2 are shown

We further genotyped the *B. mallei* strain from these three PCR positive samples. After a pre-amplification step to increase the amount of template (using the Perfecta® pre-amplification kit (Quantabio) and the corresponding set of primers), DNA samples were analyzed by PCR-HRM [3]. The panel of 15 markers was used to classify the *B. mallei* strains into one of the three lineages (L1 to L3) and the branches, sub-branches, and groups to which they relate. All samples corresponded to the L2B2sB2branch, which includes *B. mallei* strains circulating in India and Pakistan. A recent study, using four new SNP markers, classified *B. mallei* strains from India and Pakistan into two small and large subgroups [5]. This new set of markers were investigated in the three positive Nepalese samples from Lalitpur (L/157) and Banke (B/113 and B/120). The results indicated that all samples clustered in the India_group 2 (large), which includes most of the Indian strains typed so far with this new set [5], all originating from the states of Uttar Pradesh and Haryana, in Northern India (Fig. 3). The origin of the India_group 1 (small) strains is not clearly defined at this time.

## Discussion and conclusions

Recently Adhikari and colleagues [11] warned about the potential risks of glanders outbreaks in Nepal due to the re-emergence of the disease in neighboring Indian states [12–14], and the unrestricted movement of equids between the two countries. Most equids from India passed through the Nepalgunj quarantine office, the closest to India’s Uttar Pradesh region [11], where glanders cases are regularly reported [12–14]. Our epidemiological investigation indicates the equids were imported from Uttar Pradesh through uncontrolled routes, as there is about 1,770 kilometers of open border between India and Nepal. Further, there is seasonal migration of horses and mules from far western part of Nepal to western and central part and back to the India’s Uttar Pradesh region. Most of the equids in Nepal (an estimated number of 59,762; Ministry of Agriculture and Livestock Development (MOALD), 2019/2020) are used in the brick industry for transportation of bricks, goods and pulling carts. Equids are also popular in the tourism industry for transport of goods by trekkers. Horses are only vehicles for transport of goods and humans in high hill areas where mechanical vehicles cannot be used [15].

In May 2021, Nepal notified to OIE its first outbreak of glanders. Until now, no policy for prevention and control of this disease was implemented. The confirmation of glanders cases, detected in central and mid-western parts of Nepal and reported in this study, should prompt a national surveillance programme and enhanced border control measures. In general, the glanders situation in Asia is very poorly documented and strict measures are necessary to control this re-emergent disease.

## Data Availability

All data generated or analyzed during this study are included in this published article. The datasets generated during and/or analysed during the current study are available from the corresponding author.

## Abbreviations

CFT: Complement fixation test
DNA: Desoxyribose Nucleic Acid
iELISA: Indirect ELISA
ELISA: Enzyme-Linked Immuno Assay
HRM-PCR: High Resolution Melting-PCR
PCR: Polymerase Chain Reaction
SNP: Single Nucleotide Polymorphism
